# Unraveling oxidative stress response in the cestode parasite *Echinococcus granulosus*

**DOI:** 10.1038/s41598-019-52456-3

**Published:** 2019-11-04

**Authors:** Martín Cancela, Jéssica A. Paes, Hercules Moura, John R. Barr, Arnaldo Zaha, Henrique B. Ferreira

**Affiliations:** 1 Laboratório de Genômica Estrutural e Funcional, Centro de Biotecnologia, Universidade Federal do Rio Grande do Sul, UFRGS, Porto Alegre, Brazil; 2 Laboratório de Biologia Molecular de Cestódeos, Centro de Biotecnologia, Universidade Federal do Rio Grande do Sul, UFRGS, Porto Alegre, Brazil; 3 Programa de Pós-Graduação em Biologia Celular e Molecular, Centro de Biotecnologia, UFRGS, Porto Alegre, Brazil; 4 Biological Mass Spectrometry Laboratory, Clinical Chemistry Branch, Division of Laboratory Sciences, National Center for Environmental Health, Centers for Disease Control and Prevention, Atlanta, GA USA; 5 Departamento de Biologia Molecular e Biotecnologia, Instituto de Biociências, UFRGS, Porto Alegre, Brazil

**Keywords:** Apoptosis, Mechanisms of disease, Proteomics

## Abstract

Cystic hydatid disease (CHD) is a worldwide neglected zoonotic disease caused by *Echinococcus granulosus*. The parasite is well adapted to its host by producing protective molecules that modulate host immune response. An unexplored issue associated with the parasite’s persistence in its host is how the organism can survive the oxidative stress resulting from parasite endogenous metabolism and host defenses. Here, we used hydrogen peroxide (H_2_O_2_) to induce oxidative stress in *E. granulosus* protoescoleces (PSCs) to identify molecular pathways and antioxidant responses during H_2_O_2_ exposure. Using proteomics, we identified 550 unique proteins; including 474 in H_2_O_2_-exposed PSCs (H-PSCs) samples and 515 in non-exposed PSCs (C-PSCs) samples. Larger amounts of antioxidant proteins, including GSTs and novel carbonyl detoxifying enzymes, such as aldo-keto reductase and carbonyl reductase, were detected after H_2_O_2_ exposure. Increased concentrations of caspase-3 and cathepsin-D proteases and components of the 26S proteasome were also detected in H-PSCs. Reduction of lamin-B and other caspase-substrate, such as filamin, in H-PSCs suggested that molecular events related to early apoptosis were also induced. We present data that describe proteins expressed in response to oxidative stress in a metazoan parasite, including novel antioxidant enzymes and targets with potential application to treatment and prevention of CHD.

## Introduction

*Echinococcus granulosus* is the causative agent of cystic hydatid disease (CHD), a neglected zoonosis that harms human health and livestock farming worldwide^1–3^. The metacestode or hydatid cyst is the larval stage of *E. granulosus*, that develops in lungs and liver of mammalian intermediate hosts and is responsible for the pathogenesis of the infected organ and adjacent tissues^4^. The hydatid cyst is a fluid-filled cavity delimited by a carbohydrate-rich acellular laminar layer and an inner germinal layer^5^. The germinal layer is composed of stem cells capable of giving rise to the pre-adult forms or protoscoleces (PSCs). The parasite is well-adapted to its intermediate host, where it can persist for decades^6^, surviving and growing despite the adverse host responses. To secure this, the parasite developed mechanisms to subvert the host immune response^7,8^.

Antioxidant defenses are essential to combat reactive oxygen and nitrogen species (ROS and RNS, respectively) produced during host immune response and intracellular oxidative metabolism. ROS and RNS are harmful to tissue components because they can damage proteins, lipids, carbohydrates, and DNA, altering their functions^9^. For this reason, unicellular and multicellular organisms have developed non-enzymatic and enzymatic machineries, which include a repertoire of molecules to manage oxidative stress, such as glutathione and Cys-rich oligopeptides (for non-enzymatic mechanisms), and superoxide dismutases and peroxiredoxins (for enzymatic mechanisms)^10^.

A well-characterized antioxidant defense in *E. granulosus* is the linked thioredoxin-glutathione system, including the redox-associated proteins thioredoxin glutathione reductase, thioredoxin peroxidase, thioredoxin, glutathione, and glutaredoxin^11,12^. Other detoxifying enzymes, including members of the glutathione-S-transferase (GST) family, have also been reported in *E. granulosus*^13,14^. Despite the availability of genome data from various taenid species^15^, little is known about how parasitic flatworms respond to oxidative stress.

Hydrogen peroxide (H_2_O_2_) is an oxidant molecule previously used in studies as a model for oxidative stress damage^9^, wound repair^16,17^, and signaling^18^, and as an apoptosis inductor^19,20^. In *E. granulosus*, H_2_O_2_ was used as a prophylactic method to kill PSCs inside the hydatid cyst^21^, and *in vitro* exposure to H_2_O_2_ induced PSC apoptosis after 8 h of treatment^22^. Moreover, in *Echinococcus multilocularis*, the etiological agent of alveolar echinococcosis, H_2_O_2_ exposure induced expression of a tumor suppressor protein p53 homologue (Emp53), and apoptosis in metacestode vesicles cultured *in vitro*^23^. However, no study has assessed the molecular pathways activated upon oxidative stress response in the *Echinococcus* spp.

To unravel molecular mechanisms related to oxidative stress response in *E. granulosus*, we performed a comparative proteomic study between PSCs exposed and not exposed to H_2_O_2_. Overall, we identified 550 protein species, including proteins with differential abundance in response to H_2_O_2_. Up-regulation of antioxidant enzymes, including GSTs, along with novel carbonyl detoxifying enzymes, such as aldo-keto reductase and carbonyl reductase, occurred after H_2_O_2_ exposure. Other proteins that are targets of the proteolytic pathway of apoptotic cell death were down-regulated in H_2_O_2_-exposed PSCs. Overall, our results shed light on novel antioxidant mechanisms and cellular stress response in *E. granulosus*.

## Results

### MS-based proteomics analyses of PSC treated or not with H_2_O_2_

**Figure 1:**
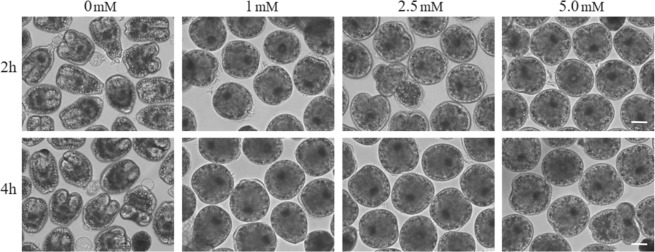
*E*. *granulosus* PSCs incubated with H_2_O_2_. PSCs were cultured with different H_2_O_2_ concentrations (0, 1.0, 2.5, and 5.0 mM) and incubation times (2 h and 4 h). The figure represents the results obtained from two biological replicates (PSCs from two different cysts). Scale bar: 40 μm.

To initially assess the overall sensitivity of PSCs to oxidative stress, independent PSC cultures were treated with different H_2_O_2_ concentrations of 1.0, 2.5, and 5.0 mM and observed after 2 h and 4 h of treatment (Fig. 1). After 2 and 4 h incubation, it was observed that PSCs adopted a rounded shape in the presence of different H_2_O_2_ concentrations, but no viability changes were noticed in comparison to the corresponding non-treated control cultures. Since previous data showed apoptosis induction in *E. multilocularis* vesicles treated with 5 mM H_2_O_2_ for 4 h, we chose a milder treatment with 2.5 mM H_2_O_2_ concentration for 2 h to allow the detection of proteins induced at earlier stages of the oxidative stress response.

Protein samples obtained from two *E. granulosus* biological replicates for each treatment (tests and control) and analyzed using 12% SDS-PAGE showed a complex pattern of proteins ranging from 10 to >225-kDa (Fig. S1). Biological replicates from control (C-PSCs) and H_2_O_2_-treated PSCs (H-PSCs) had nearly identical electrophoretic profiles.

**Figure 2:**
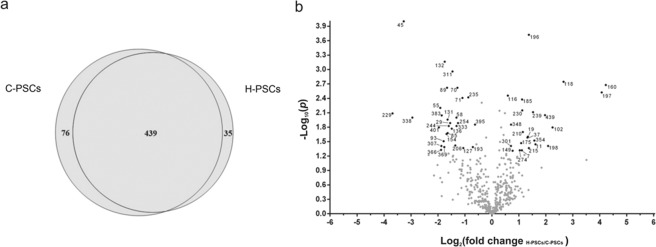
Overview of the proteins identified in the C-PSCs and H-PSCs samples. (**a**) The numbers of proteins exclusively detected in each sample or shared between them are indicated in the diagram. Only proteins identified by at least two peptides and present in the two biological replicates were considered for analysis. (**b**) Volcano plot of proteins shared between C-PSCs and H-PSCs, with significant differences between samples. Proteins with a *p* value < 0.05 (−log_10_ = 1.3) and a fold-change (FC) > 1.5 (log_2_ = 0.5) were considered differentially abundant between H-PSC and C-PSC by both statistical and FC parameters and are represented by black dots and identified by their numbering in Table S4. Proteins without significant differences in abundance between samples according to the criteria above are shown as grey dots.

LC-MS/MS analysis of the protein extracts from H-PSCs and C-PSCs in each experimental condition identified both shared and exclusive proteins. Reproducibility between replicates was assured, considering as valid only proteins identified in both biological replicates. For quantification, mass spectrometry data from the three technical replicates for each validated protein were condensed as an average of spectral counts. Overall, 550 unique proteins were identified, 474 in H-PSCs and 515 in C-PSCs (Tables S1, S2, respectively). Table S3 shows detailed peptide identification data. A total of 439 proteins were found in both H-PSCs and C-PSCs samples, while 76 proteins were exclusively found in C-PSCs and 35 were exclusive to the H-PSCs (Fig. 2a).

### Up- and down-regulated proteins in response to H_2_O_2_

Quantitative analyses using normalized spectral abundance factor (NSAF) values of the 439 proteins shared between H-PSCs and C-PSCs revealed that 52 proteins showed quantitative differences among treated and control groups, with *P* < 0.05 and fold change >1.5 (Fig. 2b). Among these 52 differentially represented proteins, 29 were down-regulated (Table 1) and 23 were up-regulated in H_2_O_2_-treated PSCs (Table 2).

**Table 1:** Proteins up-regulated in C-PSCs samples.

Protein name	Accession code^a^	*p*-value (*t*-test)	Quantitative values (NSAF)		Fold change (FC)^b^
			C-PSCs	H-PSCs	
Acidic leucine rich nuclear phosphoprotein	EgrG_001104800	0.013	0.0488735	0.0167205	2.92
Aldehyde dehydrogenase mitochondrial	EgrG_000389100	0.0001	0.062014	0.00645025	9.61
Aminopeptidase	EgrG_001105200	0.0063	0.0253525	0.0067329	3.76
Aminotransferase class III (Ornithine aminotransferase)	EgrG_001032200	0.01	0.097817	0.039335	2.48
Asparaginyl tRNA synthetase cytoplasmic	EgrG_000348600	0.0024	0.097845	0.040421	2.42
Aspartate aminotransferase	EGR_07719	0.0039	0.087438	0.0411095	2.12
Betaine aldehyde dehydrogenase	EgrG_000904200	0.021	0.0259865	0.00833905	3.11
Calcium-transporting ATPase	EGR_06085	0.0024	0.00911835	0.002885	3.16
Calnexin	EGR_06707	0.031	0.0343755	0.00996025	3.45
Cysteine and glycine rich protein 1 (Cysteine and glycine-rich protein)	EgrG_000893500	0.043	0.107258	0.0514965	2.08
Cytoplasmic dynein 1 heavy chain	EGR_01376	0.011	0.0028364	0.000907625	3.12
Dehydrogenase/reductase SDR family member	EGR_07430	0.00069	0.029967	0.0089241	3.35
Dipeptidyl peptidase 3 (Dipeptidyl peptidase III)	EgrG_001028100	0.017	0.024112	0.0086011	2.80
Endophilin B2	EgrG_000060900	0.022	0.129905	0.0405845	3.20
Glutathione S-transferase	EGR_07276	0.041	0.24975	0.153215	1.63
Gynecophoral canal protein	EgrG_000824400	0.038	0.0236955	0.00926185	2.55
Lamin-B2	EGR_02565	0.0082	0.021778	0.0016971	12.83
LIM zinc bindingdomain containing protein	EgrG_000539800	0.0038	0.0350835	0.019154	1.83
Major vault protein	EgrG_000142500	0.015	0.379795	0.12536	3.02
Mitochondrial dicarboxylate carrier	EgrG_000595000	0.013	0.0510985	0.021425	2.38
Purine nucleoside phosphorylase (PNP)	EgrG_000622900	0.039	0.098687	0.0269765	3.65
Pyrroline 5 carboxylate reductase	EgrG_000233100	0.0011	0.0099199	0.00361065	2.74
Serine protease inhibitor	EgrG_001193100	0.015	0.060694	0.0244525	2.48
Seryl tRNA Synthetase	EgrG_001197300	0.01	0.0484465	0.0062857	7.70
Tegumental protein	EGR_08411	0.047	0.036927	0.01000965	3.69
Telomerase protein component 1	EgrG_001036600	0.041	0.022413	0.0065969	3.39
Transaldolase	EGR_10111	0.009	0.0749545	0.0206575	3.62
Troponin I 4	EGR_06361	0.014	0.0929875	0.060291	1.54
Tubulin polymerization promoting protein family	EgrG_000096900	0.016	0.073331	0.01876	3.90

^a^Protein accession codes were retrieved from *E. granulosus* genome annotation available on WormBase ParaSite (http://www.parasite.wormbase.org/).

^b^Fold changes were based on NSAF values from ‘C-PSCs’ divided by those of ‘H-PSCs’.

Among the more abundant proteins in response to H_2_O_2_, we found enzymes related to oxido-reductase activity (estradiol 17 beta-dehydrogenase, protein disulfide isomerase), glycerol metabolism (glycerol-3-phosphate dehydrogenase), proteolytical activity (cathepsin D, 26S proteasome subunit), stress response (heat shock protein 70), and basement membrane component (collagen alpha-1 type IV and XI) (Table 2). Enzymes with the highest fold change (FC) are related to stress response (Fig. 2b). Among them are enzymes with different functions, such as removal of reactive carbonyl groups produced during oxidative stress (estradiol 17 beta-dehydrogenase, FC = 18.7), hyperosmotic stress response (glycerol-3-phosphate dehydrogenase, FC = 16.9), matrix cellular remodeling (collagen alpha-1(XI) chain, FC = 6.8), and protein degradation process and cathepsin D, FC = 4.8). Interestingly, previously described antioxidant enzymes, like superoxide dismutase and components of the thioredoxin system, were not differentially represented between H-PSCs and C-PSCs.

**Table 2:** Proteins up-regulated in H-PSCs samples.

Protein name	Accession code^a^	*p*-value (*t*-test)	Quantitative values (NSAF)		Fold change (FC)^b^
			C-PSCs	H-PSCs	
26S proteasome non-ATPase regulatory subunit	EgrG_000736900	0.048	0.00686595	0.0148795	2.17
40S ribosomal protein S13	EgrG_000856900	0.036	0.019052	0.058467	3.06
6 phosphogluconolactonase	EgrG_000445200	0.025	0.0117905	0.029843	2.53
Adenosylhomocysteinase	EGR_05478	0.026	0.0077762	0.0193915	2.49
Cathepsin d lysosomal aspartyl protease	EgrG_000970500	0.016	0.0158975	0.075669	4.76
Collagen alpha-1(IV) chain	EGR_08512	0.0035	0.0142325	0.021473	1.50
Collagen alpha-1(XXIV) chain	EGR_03871	0.0018	0.0014417	0.0090935	6.30
Elongation factor 1-alpha	EgrG_000982200	0.049	0.091483	0.15607	1.70
Estradiol 17 beta-dehydrogenase	EGR_09847	0.0021	0.0098417	0.18411	18.70
Fatty acid amide hydrolase 1	EgrG_000743700	0.034	0.014514	0.03086	2.12
GDP L fucose synthase	EgrG_000476900	0.0042	0.0196555	0.0427775	2.17
Glycerol-3-phosphate dehydrogenase NAD	EgrG_000686100	0.00019	0.010596	0.027413	2.58
Glycerol-3-phosphate dehydrogenase NAD(+)	EGR_09089	0.003	0.00262725	0.044269	16.84
Glycogen debranching enzyme	EgrG_000644500	0.039	0.00428925	0.018231	4.25
Heat shock protein 105	EgrG_000917000	0.02	0.018896	0.0419425	2.21
Histone	EgrG_002016600	0.043	0.0710695	0.18414	2.59
Large subunit ribosomal protein 23	EgrG_000954600	0.0071	0.0174355	0.038076	2.18
Long chain fatty acid coenzyme A ligase 4	EgrG_000376500	0.0077	0.0084074	0.0242155	2.88
Nucleoside diphosphate kinase	EGR_05582	0.048	0.074672	0.152835	2.04
Protein disulfide-isomerase	EGR_08944	0.039	0.031717	0.051831	1.63
Splicing factor U2AF subunit (U2 small nuclear RNA auxiliary factor 2)	EgrG_000625400	0.014	0.0037978	0.00622035	1.63
Succinyl-CoA synthetase subunit alpha	EGR_08424	0.03	0.015809	0.047168	2.98
Zinc phosphodiesterase elac protein 1	EgrG_000149350	0.0089	0.00665745	0.0261045	3.92

^a^Protein accession codes were retrieved from *E. granulosus* genome annotation available on WormBase ParaSite (http://www.parasite.wormbase.org/).

^b^Fold changes were based on NSAF values from ‘H-PSC’ divided by those of ‘C-PSC’.

Among the 29 proteins down-regulated in H-PSCs (up-regulated in C-PSCs), we found structural proteins (lamin-B2, dynein-1 heavy chain, troponin I), proteins with oxido-reductase activity (dehydrogenase/reductase, aldehyde dehydrogenase, pyrroline 5-carboxylate reductase), inhibitors and proteolytic enzymes (serine proteinase inhibitor, dipeptidyl peptidase 3, aminopeptidase), and those involved in protein synthesis (asparaginyl and seryl tRNA synthetases) (Table 1). According to the volcano plot analysis, the most down-regulated proteins were lamin B-2 (FC = 12.8), aldehyde dehydrogenase (FC = 9.6), and seryl tRNA syntethase (FC = 7.7) (Fig. 2b). Two protein components of the vault complex (major vault protein and telomerase protein component 1) were also down-regulated (FC ~3) in H-PSCs.

### Proteins exclusively detected in H-PSCs and C-PSCs

We identified 76 proteins exclusive to C-PSCs and 35 proteins exclusive to H-PSCs (Table S3). In H-PSCs, these proteins included two GST detoxifying enzymes (EGR_07274 and EGR_09218), caspase-3 related to apoptosis (EgrG_000462900), proteolytic enzymes related to proteasome (EGR_04682, EgrG_000223600 and EGR_05037), and other carbonyl detoxifying enzymes, such as carbonyl reductase 1 (EgrG_000113500) and aldo-keto reductase family 1 (EgrG_000156300). Conversely, in C-PSCs, we found many expressed conserved proteins with unknown function (e.g., EgrG_000087900, EgrG_000097300, EgrG_000124000), proteins related with protein synthesis, such as ribosomal proteins and aminoacyl-tRNA synthetases (Table S3) and some cytoskeletal proteins (filamins, talin, tubulin-β, tropomyosin).

### Functional annotation and gene ontology (GO) term enrichment analyses

To unravel molecular pathways associated with oxidative stress response, we performed functional annotation for proteins differentially represented in H-PSCs (58, being 35 exclusive plus 23 up-regulated) and C-PSCs (105, being 76 exclusive plus 29 up-regulated). Using gene functional categories defined by the gene ontology and GO terms were summarized by REVIGO. Six REVIGO category clusters were enriched in H-PSCs samples: metabolic process, glycerol-3-phosphate catabolic process, oxidation-reduction process, carbohydrate metabolic process, organic substance metabolic process, and primary metabolic process. Eight REVIGO category clusters were enriched in C-PSCs samples. The top five among those eight included tricarboxylic acid metabolic process, cellular component assembly involved in morphogenesis, mitotic cell cycle process, tRNA aminoacylation for protein translation, and cytoskeleton organization.

### GST immunolocalization in PSC under H_2_O_2_ exposure

**Figure 3:**
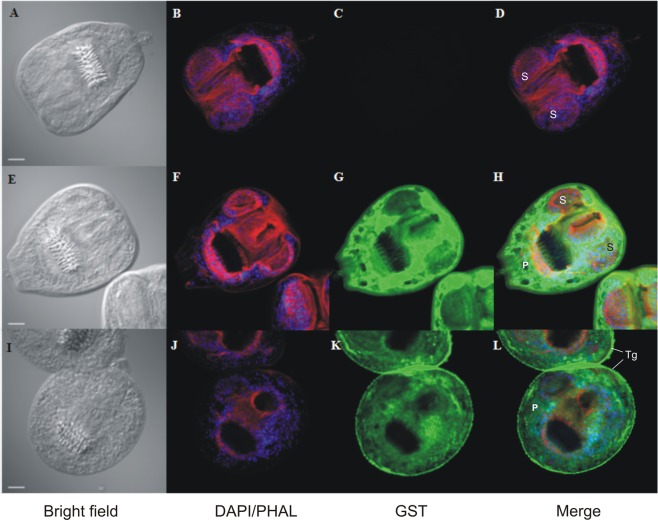
*In toto* immunolocalization of GST during PSCs oxidative stress response. PSCs were incubated in RPMI-10% FBS (C-PSCs) (A to H) or RPMI-10% FBS supplemented with 2.5 mM H_2_O_2_ (H-PSCs) (I to L). After PFA fixing, PSCs were incubated with anti-GST antibody (G and K) (GST) (green) and counterstained with DAPI-phalloidin (DAPI/PHAL) (see Materials and Methods section). Negative control consisted of omission of primary antibody (C). A, E, and I show the bright field and merge of the three channels in D, H and L. Scale bar = 20 μm. P: parenchyma: S: sucker: Tg: tegument.

We performed a whole-mount immunohistofluorescence (WMIF) using a monoclonal antibody raised against Sj28GST in H-PSCs and C-PSCs. This antibody recognized a specific 27-kDa band, as expected for EgGST isoforms, in western blot assays using PSC soluble extract (data not shown). In WMIF experiments, the anti-Sj28GST allowed to detect differences in GST localization between H-PSCs and C-PSCs. In C-PSCs, GST was localized in cytoplasmic and parenchymal tissues. In H-PSCs, GST showed strong reactivity in the tegumental surface of protoescoleces (Fig. 3). This correlated with the differential GST expression between C-PSCs and H-PSCs and suggested a possible role for different EgGSTs in the protection of PSCs against oxidative damage induced by H_2_O_2_.

## Discussion

During the *E. granulosus* life cycle, onchospheres, hydatid cysts and PSCs are exposed to host and endogenous toxic compounds. As a consequence, the parasite makes use of different molecular mechanisms to eliminate them. For instance, PSCs are constantly exposed to ROS and RNS during infection, either within the hydatid cyst or upon their release in the definitive (primary infection) or intermediary hosts (secondary infections)^24^. In primary infection, as PSC passes though the digestive tract, it is activated by the action of pepsin and H+, at the stomach, and by bile salts, at the small intestine. During this process, PSCs evaginate and fix themselves to the gut mucosa, where they develop into adult tapeworms. At the intestine, ROS and RNS can be produced by phagocytic cells as part of the mucosal immune response and also by the epithelium and microbiota^25^. In secondary infections, caused by cyst content leakage or rupture, PSCs and additional cyst components can also activate host defenses, including phagocytic cells that can produce H_2_O_2_^26^, which is toxic for PSCs^21^. Despite this hostile host environment, PSCs are able to survive and differentiate into secondary hydatid cysts^27^, relying for that on the production of antioxidant (AOX) molecules and enzymes that prevent oxidative damage to macromolecules.

In our study, we used hydrogen peroxide to induce oxidative stress in PSCs of the platyhelminth *E. granulosus*, which represents a condition found by the parasite when it infects mammalian hosts. We used mass spectrometry proteomics workflow to identify proteins and molecular pathways associated with oxidative stress response in the parasitic platyhelminth *E. granulosus*.

**Figure 4:**
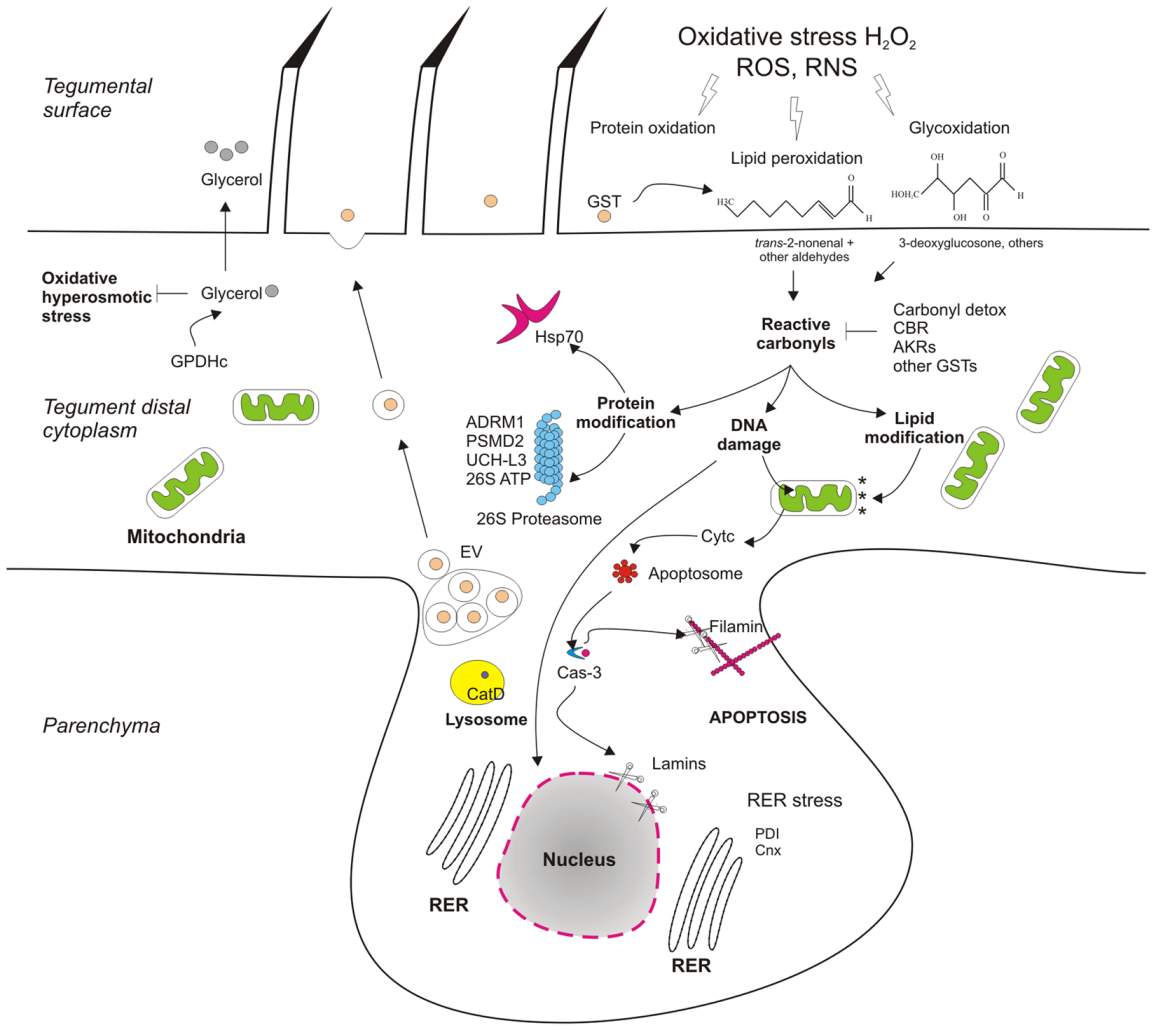
Molecular pathway linked to oxidative stress response in *E. granulosus* PSCs. Exposure to H_2_O_2_ and other ROS/RNS can cause macromolecule oxidation at the tegumental surface of PSC leading to reactive carbonyls production. Expression of GST at the parasite surface can contribute with the detoxification of some toxic aldehydes (i.e., *trans*-2-nonenal). Other carbonyl detoxifying enzymes (AKRs, CBRs, and GSTs) are up-regulated to prevent lipid, protein, and DNA damage. Protein modification can cause protein aggregation that can be inhibited by Hsp70 chaperone or degraded by targeting ubiquitinated proteins to 26S proteasome (ADRM1, PSMD2, UCH-L3, and 26S ATP). Damaged mitochondrial membrane and nuclear/mitochondrial DNA could be responsible for initial events of apoptosis, inducing caspase-3 activation and cleavage of protein substrates such as filamins and lamins. ER stress induced by oxidative stress can also be associated with PSC apoptosis. Glycerol production can protect cells from hyperosmotic and oxidative stress. EV: extracellular vesicle, Cytc: cytochrome c, CatD: cathepsin D, Cnx: calnexin, PDI: protein disulfide isomerase.

Our proteomic analysis identified many PSC proteins whose relative abundances were altered in response to H_2_O_2_. The generated data allowed to propose molecular pathways and functions that are activated upon *E. granulosus* PSCs exposure to H_2_O_2_ and likely contribute to protect the parasite against oxidative damage (Fig. 4). Unexpectedly, previously characterized antioxidant enzymes, like superoxide dismutase^28^ and components of the thioredoxin system^11^ were not up-regulated in H-PSCs. A possible explanation for that would be that we only analyzed PSCs collected after 2 h of exposure to H_2_O_2_. Possibly, these enzymes are not the main players in AOX response at this time point.

One of the major detoxification systems in helminths includes various isoforms of GSTs^29–33^. GSTs are multifunctional enzymes that enable cellular detoxification of endogenous and exogenous toxic chemicals (xenobiotics) by catalyzing their conjugation to glutathione. A previous report showed that *E. granulosus* expresses at least three GSTs isoforms, named EgGST1-3^32^. Interestingly, different EgGST isoforms can form heterodimers *in vitro*^34^ and this would be important to increase functional diversification. Overexpression of EgGST1 was observed after PSC incubation with the GST inducer phenobarbital^13^. In our study, we found two EgGSTs up-regulated in response to H_2_O_2._ EgGST1 and a fourth, previously uncharacterized EgGST in H-PSCs indicate that different *E. granulosus* GST isoforms are induced in response to different stressor agents. The two up-regulated EgGSTs identified here might protect PSCs against lipid peroxidation, generated by H_2_O_2_ and other ROS produced by the host^35^. Using an *S. japonicum* anti-GST antibody, we found immunoreactivity at the tegumental surface of PSC exposed to H_2_O_2_. In contrast, control PSCs showed a strong GST immunoreactivity in internal parenchymal tissues. These data suggest that GSTs isoforms are synthetized in cells lining the tegumental surface during oxidative stress, protecting PSC from exogenous oxidants. In the parasitic trematode *Clonorchis sinensis*, differential activation of GST isoforms has occurred in response to bile salts or oxidative stress, with up-regulation of secreted isoforms upon oxidative stress^36,37^.

Many carbonyls (aldehydes and ketones) produced after oxidative stress are highly reactive, leading to cellular damage through DNA adduct formation and protein and lipids modifications^38^. ROS and RNS can cause oxidation of macromolecules to reactive carbonyls. To counter carbonyl stress, cells express a group of enzymes involved in carbonyl metabolisms, including aldo-keto reductases (AKRs) and carbonyl reductases (CBRs)^39^. We found an estradiol-17 beta dehydrogenase with the highest fold change (FC ~19) and another member of the AKR family (EgrG_000156300) among the proteins up-regulated in PSCs exposed to H_2_O_2_. These enzymes belong to the aldo-keto reductase superfamily (AKR domain, pfam00248 identified). This protein superfamily is composed of >190 members distributed in 16 AKR families in humans. AKRs are present in all phyla acting as NADPH-dependent oxidoreductase enzymes with important role in reduction of aldehydes to alcohol^40^. For instance, an AKR enzyme was found as secreted by the trematode parasite *Echinostoma caproni* and assumed to contribute to withstand hostile Th1 pro-inflammatory environment induced in primary infections in mice^41^. In cancer cells, overexpression of some members of the AKR family protects against drug toxicity and apoptosis induced by reactive carbonyl^42,43^. In the parasitic protozoan *Trypanosoma cruzi*, the etiological agent of Chagas disease, *T. cruzi* AKR (TcAKR) was up-regulated in benznidazole-resistant strains^44^. Overexpression of TcAKR gene in a sensitive strain enhanced the resistance to benznidazole and reduced intracellular ROS after treatment^45^, supporting TcAKR antioxidant activity and involvement in drug metabolism. Moreover, in the protozoan *Babesia microti*, responsible for human babesiosis, AKR was up-regulated in response to oxidative stress and anti-parasitic drugs, supporting the relevance and conserved functions of AKRs against oxidative damage and drug response^46^. The induction of some members of the AKR family upon PSCs exposure to H_2_O_2_ suggests that these enzymes play an important role in detoxifying reactive carbonyls, a protective response against ROS in *E. granulosus*. Carbonyl reductase 1, another carbonyl detoxifying enzyme, was up-regulated in H-PSCs. This enzyme belongs to the NADPH-dependent short-chain dehydrogenase/reductase (SDR) superfamily. Carbonyl reductase 1 catalyzes the reduction of various carbonyl compounds, including endogenous aliphatic aldehydes and ketones and xenobiotic quinones^47^. Human carbonyl reductase 1 plays an important role in neuronal^48^ and cancer cell survival^49^ by decreasing oxidative stress and resistance to apoptosis. It protects cells against lipid peroxidation by reducing carbonyl aldehydes^50^. Moreover, human carbonyl reductase 3 is induced during pro-inflammatory stimuli and acts as a sensor of oxidative stress^51^. In the trematode parasite *F. hepatica*, the causative agent of fasciolosis, two xenobiotic metabolizing enzymes (carbonyl reductase and GST) showed an increased activity after *in vivo* treatment with the anthelminthic drug triclabendazole, suggesting a detoxifying function for this enzyme^52^. In *E. granulosus* a high level of carbonyl reductase in PSCs exposed to H_2_O_2_ might act to detoxify reactive carbonyl, preventing macromolecules damage. Our data show the first strong evidence on the importance of this family of carbonyl detoxifying enzymes to combat oxidative stress in helminth parasites.

Cellular damage caused by oxidative stress can induce cell death by different pathways, including programmed cell death. Previous work showed that H_2_O_2_ induced apoptosis of *E. multilocularis* metacestode vesicles^53^, but the molecular aspect of oxidative stress that lead to cell death are yet unknown. In our study, we found evidence of the molecular event related to cell death of PSC after H_2_O_2_ treatment. Lamins (type-A and B) are components of the nuclear lamina and are major structural proteins. These proteins are located at the inner membrane of nuclear envelope and play important functions in nuclear architecture and maintenance of chromosome integrity by ensuring proper spindle assembly^54^. In the present study, we found lamin-B down-regulated in PSC after H_2_O_2_ exposure (FC = 6). During apoptosis, lamins are degraded by caspases and considered to be among the initial nuclear target cleaved during the apoptotic process^55,56^. In our study, caspase-3 was exclusively detected in H-PSCs, suggesting that apoptotic pathway and cleavage of protein targets are active after exposure to H_2_O_2_.

Proteolytic enzymes other than caspases have been reported in apoptotic execution^57,58^. We found a cathepsin D lysosomal aspartic endopeptidase up-regulated in H-PSCs (FC = 4.8). Cathepsin D enzyme is important not only for protein catabolism but also for regulating many biological functions^59^, including programmed cell death induced by apoptotic stimuli, such as ROS (e.g., H_2_O_2_)^60,61^ and cell senescence^62^. Pro-apoptotic activity of cathepsin D is related to the post-translational modification of cytosolic Bax and translocation to mitochondrial membrane in oxidative stress-treated cells^63^. This event results in loss of mitochondrial membrane stability and apoptosis. Other caspase substrates found down-regulated in H-PSCs are filamins, proteins involved in cell adhesion, which contributes to mechanical stability of the cell^64^. Previous work showed that filamins protect cells against force-induced apoptosis and also are a caspase substrate^65^, suggesting that lower expression of filamins in H-PSCs is related to active apoptotic pathway and cellular changes induced by H_2_O_2_.

Protein oxidation can cause protein misfolding and aggregation, with harmful effect on cell physiology. Thus, mechanisms preventing or retarding accumulations of non-functional proteins can help cells survive. Heat-shock proteins (HSPs) are a family of highly conserved proteins in all domains of life^66^. Many HSPs have chaperone activity, assisting folding of nascent proteins and refolding, maintaining protein homeostasis in physiological and stress conditions^67^. In our study, we found up-regulation of *E. granulosus* 70-kDa HSP (HSP70) in PSCs exposed to H_2_O_2_. Previous data showed that HSP70 induced during oxidative stress conferred protection against apoptotic death by sequestering protein aggregates^68^ and stabilizing pro-apoptotic proteins^69^. Additional HSP70 anti-oxidant activity was associated to the ability to attenuate membrane lipid peroxidation during oxidative stress induced by H_2_O_2_^70^. Protein-disulfide-isomerase (PDI) is a member of the thioredoxin superfamily of redox proteins located at the endoplasmic reticulum lumen. PDI catalyzes disulfide formation and reduction (oxidoreductase activity) and the rearrangement of incorrect disulfide (isomerase activity), assisting folding and maturation of newly synthetized proteins, redox cell signaling, and homeostasis^71^. Because oxidative stress profoundly affects cysteine oxidation within proteins^72^, *E. granulosus* PDI up-regulation after exposure to 2.5 mM H_2_O_2_ could be important to maintain the protein redox status to prevent misfolding and aggregation. Although PDI was originally identified in the endoplasmic reticulum (ER) lumen, this enzyme was also detected on the cell surface of mammalian cells and in the secretion of various parasitic helminths (*Clonorchis sinensis*^73^, *Fasciola hepatica*^74^, and *Angiostrongylus cantonensis*^75^). HSP, together with other chaperones, could help restore protein folding during cytotoxic or proteotoxic stress, allowing parasite survival in stressing conditions.

Calnexin (Cnx), another ER resident chaperone, is an integral membrane protein that assists protein folding and quality control of proteins through the secretory pathway^76^. During oxidative stress, protein oxidation can cause protein misfolding and accumulation in the ER lumen, leading to a condition termed ER stress^77^. Down-regulation of the Cnx chaperone by microRNA or RNAi silencing has been implicated in ER stress and induction of apoptosis in mammalian cells^78–80^. Because PSC exposed to H_2_O_2_ showed a significant decrease in Cnx (FC = 3.4), this protein might be important in ER stress-induced apoptosis after ROS stimuli in *E. granulosus*. In line with our results, a recent work evaluated the cytotoxic effect of arsenic trioxide (As_2_O_3_) in *E. granulosus* PSCs. The authors showed that As_2_O_3_ cytotoxicity was due to elevation of ROS production and induction of ER stress-induced apoptosis^81^.

Another way to prevent accumulation of misfolded or oxidized proteins during oxidative stress is through degradation by the ubiquitin-proteasome system (UPS)^82^. Up-regulation of proteasome subunits in response to oxidative stress has occurred in yeast and mammalian cells^83^. In accordance with this, up-regulation of many protein components of the UPS system were observed in H-PSCs. Among these proteins, we found two ubiquitin-binding proteasome subunits (ADRM1 and PSMD2) that act as ubiquitin receptors, important to recruit ubiquitinated proteins to the proteasome^84^. We also observed some structural components of the 26S proteasome and ubiquitin carboxy-terminal hydrolase L3 (UCH-L3), a deubiquitinated enzyme involved in ubiquitin and ubiquitin-like nedd8 cleavage from ubiquitinated proteins^85^, up-regulated in H-PSCS. Members of the UCH family are important in the control of cell growth^86^, cell survival^87^, and in preventing proteotoxic effect of protein accumulation in mice skeletal muscles^88^. Recent work supports the importance of proteasome in regulating apoptosis by degradation of pro-apoptotic proteins^89^ as a mechanism of resistance to apoptotic cell death.

Adaptation to osmolality changes is fundamental for cellular and organism survival in different environments^90^. Hyperosmotic stress promotes water efflux, inducing cellular shrinkage, DNA and protein damage, and cell death^90^. Cell exposure to H_2_O_2_ showed a marked alteration in membrane fluidity, increasing drug permeability^91^, changes in membrane potential^26^, and cell shrinkage^92^. Production of organic osmolytes, such as glycerol, is an adaptive mechanism to circumvent this stress^93^. Glycerol-3-phosphate dehydrogenase (GPDH), an enzyme involved in glycerol production, was up-regulated under hyperosmotic stress in the yeast *Sacharomyces cerevisiae*^94^ and the free-living nematode *Caenorhabditis elegans*^95^. Transfection of Chinese hamster ovary (CHO) cells with a cytosolic isoform of GPDH (cGPDH) showed a marked cell resistance to H_2_O_2_, demonstrating the importance of this enzyme in hyperosmotic and oxidative stress protection^96^. Likewise, up-regulation of two cytosolic *E. granulosus* GPDHs occurred after H_2_O_2_ exposure. One of these cGPDHs was 19 times more abundant in H-PSCs than in untreated PSC. A mitochondrial GPDH *E. granulosus* was down-regulated in H-PSCs, suggesting that mitochondrial isoform of GPDH is involved in other functions unrelated with stress response.

In our study, we introduce several novel proteins involved in the response to oxidative stress in the helminth parasite *E. granulosus*. Our proteomic approach allowed us to identify a broad spectrum of enzymes and pathways activated in *E. granulosus* PSCs upon exposure to H_2_O_2_. Novel antioxidant enzymes related to the reactive carbonyl detoxification pathway, including AKRs and CBRs, were up-regulated in H-PSCs. AKRs and CBRs are important for preventing macromolecule damage and cell death. Up-regulation of proteolytic enzymes related to apoptotic pathways (i.e., caspase-3 and cathepsin D) and down-regulation of caspase-substrates (i.e., lamin-B and filamins) suggest that early apoptotic events were induced by H_2_O_2_ exposure. Components of the 26S proteasome involved in degradation of ubiquitinated proteins were induced in response to oxidative stress. Some of these enzymes are targets for chemotherapy in cancer and associated with protozoan drug resistance (Table 3). Repositioning of drugs for use as anti-helminth, together with the identification of novel drug targets, can contribute to the development of more effective treatment or circumvent resistance problem associated with parasitic diseases.

**Table 3:** Protein targets for drug repositioning. *E. granulosus* H-PSCs up-regulated proteins for which there are available inhibitory drugs previously used for the treatment of different infections/diseases.

Target	Drug	Mechanism of action	Disease or pathogen treatable by the drug	Ref
Glycerol-3-phosphate dehydrogenase	Anacardic acids	Non-competitive enzyme inhibition	Tumors and Bacterial pathogens	^108^
Carbonyl reductase	Biphenyl compounds	Enzyme inhibition	Breast cancer	^109^
Cathepsin D	Amprenavir^a^, Indinavir^a^, Lopinavir^a^, Nelfinavir^a^, Ritonavir^a^, Saquinavir^a^	Enzyme inhibition	Human Immunodeficiency Virus Trypanosomatids: *Leishmania* species and *T. cruzi*	^110, 111^
Estradiol 17-beta dehydrogenase	Steroidal STX1040 and non-steroidal PBRM	Enzyme inhibition	Breast cancer	^112^
Glutathione-S-transferase	Ethacrynic acid analogues^a^	Enzyme inhibition	Cancers	^113^
	Glutathione analogues: ezatiostat (TLK199)		Myelodysplastic syndrome	^114^
Proteasome subunits	Bortezomib^a^	Inhibition of p53 degradation?	Multiple myeloma	^115^
	Epoxomicin	Proteasome inhibition	*Babesia divergens*	^116^
	Carmaphycin B analogs	Proteasome inhibition	*Plasmodium falciparum*	^117^

^a^FDA-approved drugs.

## Methods

### Parasites

Hydatid cyst from *E. granulosus* sensu stricto (G1-G3 genotypes) were obtained as described before^97^. *E. granulosus*-contaminated livers and lungs were donated by a commercial slaughterhouse for use in this work. Liver and lung cysts were aseptically punctured, and PSCs were washed several times with PBS. PSCs viability were determined by trypan blue exclusion. Only batches with viability greater than 90% were used for further analysis. *E. granulosus* PSCs genotyping was carried out for each collected individual cyst, according to a previously reported protocol^98^.

### *In vitro* cultures of *E. granulosus* PSCs

*E. granulosus* sensu stricto PSCs were cultured in RPMI medium (SIGMA) supplemented with 10% fetal bovine serum (FBS) (ThermoFisher, Illinois, USA) for 2 h and 4 h at 37 °C in 5% CO_2_ (20 μL PSCs in 2 mL of medium) in the presence of different concentrations of H_2_O_2_ (Fischer, Illinois, USA). PSCs viability was determined by trypan blue exclusion. PSCs incubated without H_2_O_2_ were used as a control group.

For mass spectrometry analysis, PSCs obtained from two different cysts were independently cultured in RPMI 10% FBS (50-μL PSCs in 5 mL medium/well) for 2 h at 37 °C in the presence of 2.5 mM H_2_O_2_ or without H_2_O_2_ (control). These two biological replicates were separately harvested by sedimentation and washed four times with PBS at 37 °C. The PSC pellets were frozen in liquid nitrogen and stored at −80 °C until use.

### Preparation of PSC protein extracts for mass spectrometry analysis

For mass spectrometry analysis, protein extracts were prepared using RapiGest SF (Waters, Massachusetts, USA). Briefly, PSC pellets (obtained as described above) were suspended in 400 μL 0.1% RapiGest in 25 mM ammonium bicarbonate and sonicated for 5 cycles of 30 s with 60 s interval between pulses (30% power) with an ultrasonic homogenizer (Qsonica Sonicator, New York, USA) and then centrifuged at 20,000 x g for 30 min at 4 °C. Protein concentration in each extract was determined in the obtained supernatant fraction using Micro BCA protein assay kit (ThermoFisher, Illinois, USA) and 20 μg of each sample (H-PSCs and C-PSCs) were analyzed by 12% SDS-PAGE^99^ and stained with Coomassie’s brilliant blue R-250 (CBB-R250). Samples containing 100 μg of total protein were processed for mass spectrometry analysis according to the RapiGest SF surfactant protocol. The proteins were reduced with 5 mM DTT at 60 °C for 30 min, then alkylated using 15 mM iodoacetamide for 30 min in the dark at room temperature and incubated with mass spectrometry grade trypsin gold (Promega, Wisconsin, USA) at a ratio of 1 μg/100 μg protein. Samples were treated with 1 μg of trypsin for 4 h at 37 °C. After an additional aliquot of 1 μg of trypsin was added, samples were incubated for 16 h at 37 °C. After removing the RapiGest using 0.5% TFA, the resulting peptides were desalted using OASIS HLB cartridges (Waters, Massachusetts, USA), eluted in 50% acetonitrile and 0.1% TFA and lyophilized in a Concentrator Plus (Eppendorf, Hamburg, Germany).

### Mass spectrometry analysis

Lyophilized peptides were reconstituted in 0.1% formic acid and analyzed by LC-MS/MS. Samples were analyzed on an Orbitrap Elite tandem mass spectrometer (ThermoFisher, Illinois, USA) equipped with a nanoAcquity ultra performance liquid chromatography system (Waters, Massachusetts, USA). LC separations were performed as described previously^100^. Briefly, mobile phase solvents consisted of (solvent A) 0.1% formic acid in water and (solvent B) 0.1% formic acid in acetonitrile (Burdick & Jackson, Michigan, USA). A 8-μl volume of each sample (corresponding to 5 μg of tryptic peptides) was loaded onto a PepMap 100 C18 LC Column (03 mm × 5 mm) ThermoFisher, Illinois, USA at a flow rate of 5 μl/min. Peptides were eluted to an Easy Spray Column PepMap RSLC C18 (75 μm × 15 cm) using a nanoAcquity UPLC system (Waters Corporation, Milford, MA) and separated using a gradient elution at a flow rate of 300 nl/min. The LC gradient included a hold at 5% B for 5 min, followed by a ramp up to 35% B over 25 min, then a ramp up to 95% B in 5 min, a hold at 95% for 5 min before returning to 5% B in 5 min and re-equilibration at 5% B for 20 min. Mass spectra were collected in the data-dependent acquisition mode by scanning the mass range from mass-to-charge (m/z) 400 to 1600 at a nominal resolution setting of 60,000 for precursor ion acquisition in the Orbitrap. For the MS/MS analysis, the mass spectrometer was programmed to select the top 15 most intense ions with two or more charges. The spray voltage applied to the electrospray tip was 2.0 kV. Two biological and three technical replicates were analyzed. Each biological sample was composed of PSCs collected from one hydatid cyst.

### LC-MS/MS data analysis

MS/MS raw data were processed using msConvert tool (ProteoWizard, version 3)^101^. The peak lists were exported in Mascot generic format (.mgf). The MS/MS data were analyzed using Mascot search engine (Matrix Science, version 2.3.02) against a local *E. granulosus* database (21,764 sequences) containing the deduced amino acid sequences from the 2017 genome annotation available on WormBase ParaSite (http://www.parasite.wormbase.org/). The search parameters included a fragment ion mass tolerance of 0.6 Da and a peptide ion tolerance of 50 ppm. Carbamidomethylation was specified as a fixed modification, and oxidation of methionine was specified as a variable modification. We used Scaffold (Proteome Software Inc., version 4.4.1) to validate the peptide and protein identifications. The peptide identifications were accepted if they could be established at greater than 95.0% probability as assigned by the Peptide Prophet algorithm^102^. The protein identifications were accepted if they could be established at greater than 99% probability as assigned by the Protein Prophet algorithm^103^ and contained at least two identified peptides. The false discovery rate (decoy) was 0.0% for proteins and 0.2% for peptides. The normalized spectral abundance factor^104^ was calculated for each protein and the quantitative differences were statistically analyzed using Student’s *t*-test in Scaffold. The differences with *P*-values lower than 0.05 were considered statistically significant. The mass spectrometry proteomics data have been deposited to the ProteomeXchange Consortium via the PRIDE partner repository with the dataset identifier PXD015801.

### *In silico* functional annotation of PSC proteins identified by LC-MS/MS

Functional analyses of PSC proteins identified by LC-MS/MS were based on gene ontology (GO). The PSC identified proteins were submitted to hierarchical GO overrepresentation tests using the Cytoscape 2.6.3 plugin BiNGO 2.3^105^. Custom *E. granulosus* GO annotation files were provided by Wellcome Sanger Institute (Hinxton, UK). The ontology files were retrieved from the GO database (http://www.geneontology.org/). Annotation and ontology files were edited in-house as BiNGO input files. The hypergeometric overrepresentation tests were performed at a 0.05 level of significance, with the Benjamini-Hochberg false discovery rate multiple-testing correction. Enriched GO term lists were summarized by removing redundant GO terms using REVIGO (http://revigo.irb.hr/)^106^. The semantic similarity of the GO terms was calculated with SimRel (default allowed similarity = 0.7).

### Immunolocalization of GST in *E. granulosus* PSCs

Whole-mount immunofluorescence detection was performed as described by Fairweather *et al*., 1994^107^, with modifications. Intact PSCs were fixed for 3 h at room temperature in 4% (w/v) paraformaldehyde in PBS and then made permeable for 24 h at 4 °C in PBS containing 0.3% Triton X-100, 0.1% bovine serum albumin, and 0.2% sodium azide (P buffer). Primary *S. japonicum* anti-GST monoclonal antibody (SIGMA, Michigan, USA) was diluted 1:400 in P buffer and incubated 48 h at 4 °C. PSCs were washed 24 h at 4 °C in P buffer and then incubated 24 h at 4 °C in anti-mouse IgG conjugated to Alexa 488 (ThermoFisher, Illinois, USA) (diluted 1:400). Specimens were washed for 24 h at 4 °C. Nuclei were stained with 100 nM 4′,6-diamidino-2-phenylindole (DAPI) (Molecular Probes, Oregon, USA). Actin filaments stained with 50 nM Alexa Fluor 594-conjugated phalloidin (ThermoFisher, Illinois, USA). PSCs were examined using an Olympus FluoView 1000 confocal microscope.

## Disclaimer

References in this article to any specific commercial products, process, service, manufacturer, or company do not constitute an endorsement or a recommendation by the U.S. Government or the Centers for Disease Control and Prevention. The findings and conclusions in this report are those of the authors and do not necessarily represent the views of CDC.

## Supplementary information

Supplementary Fig 1

Table S1

Table S2

Table S3

Table S4
