# Supplementary material for: Unraveling oxidative stress response in the cestode parasite Echinococcus granulosus
Source: Sci Rep. 2019 Nov 4;9:15876. doi: 10.1038/s41598-019-52456-3 (PMC6828748; doi:10.1038/s41598-019-52456-3)
Supplement: Supplementary file 1 — Supplementary Fig 1 [file 41598_2019_52456_MOESM1_ESM.pdf]

Unraveling oxidative stress response in the cestode parasite *Echinococcus granulosus.* Martín Cancela, Jéssica A. Paes, Hercules Moura, John R. Barr, Arnaldo Zaha and Henrique B. Ferreira.

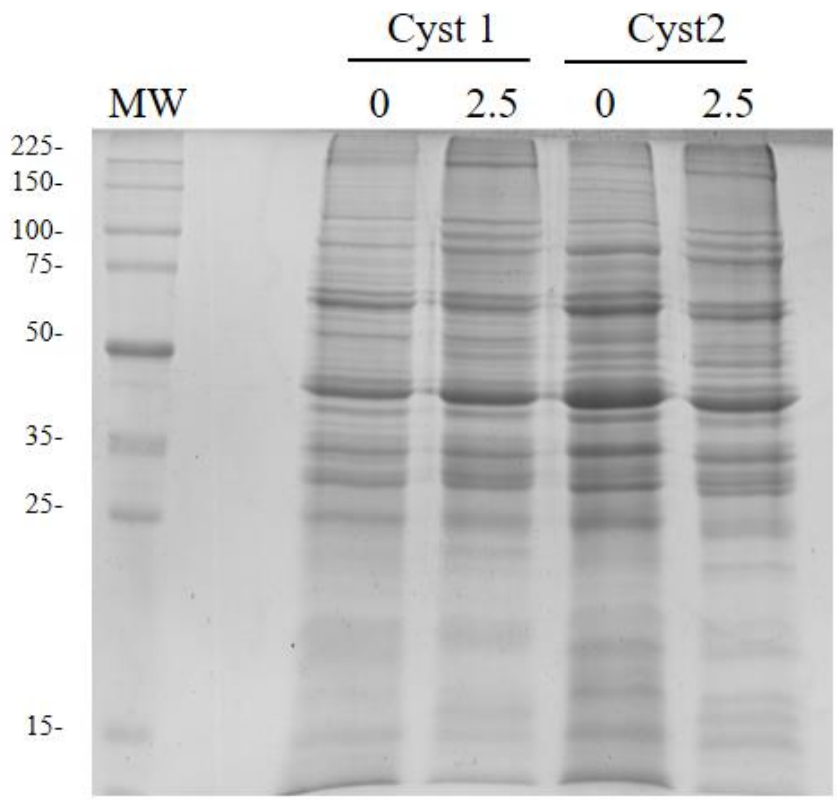

Supplementary figure 1. Protein profile of PSCs incubated with H_2O_2. PSCs from two independent cysts (biological replicates Cyst 1 and Cyst 2) were incubated in RPMI plus 10% FBS (0) and with 2.5 mM H_2O_2 (2.5) for 2 h. PSCs were then washed and processed as described in the Material and Methods section. Proteins were resolved by 12% SDS-PAGE and stained with CBB-R250. MW, molecular weight marker; 0, PSCs incubated 2 h without H_2O_2; 2.5, PSC incubated 2 h with 2.5 mM H_2O_2.
